# Push–Pull Inverter Using Amplitude Control and Frequency Tracking for Piezoelectric Transducers

**DOI:** 10.3390/mi14122147

**Published:** 2023-11-24

**Authors:** Yinghua Hu, Ming Yang, Yuanfei Zhu, Shangting Wang

**Affiliations:** School of Sensing Science and Engineering, Shanghai Jiao Tong University, Shanghai 200240, China; huyinghua@sjtu.edu.cn (Y.H.); zhuyuanfei@sjtu.edu.cn (Y.Z.); shangting@sjtu.edu.cn (S.W.)

**Keywords:** piezoelectric transducer, amplitude control, frequency tracking, push–pull inverter, driver circuit

## Abstract

Frequency tracking and amplitude control are essential for piezoelectric transducers. Frequency tracking ensures the piezoelectric transducer operates at the resonant frequency for maximum power output, and amplitude control regulates the mechanical motion of the output. This paper presents a novel driver based on a push–pull inverter for piezoelectric transducers. The proposed driver realizes the frequency tracking and amplitude control scheme by a voltage sensing bridge in the case of transformer secondary matching, guaranteeing automatic frequency tracking and precise mechanical functions regardless of environmental and load variations. The proposed scheme is verified by the ultrasonic scalpel and the ultrasonic motor (USM). The experimental results show that this scheme reduces the build-up time from 10 ms to 3 ms and loaded frequency variations from 250 Hz to 200 Hz. In addition, the amplitude control performance was further observed on USM for various loads. The overshoot is less than 5.4% under different load torques. Therefore, the proposed scheme improves the load adaptability and stability of piezoelectric transducers and promotes the application of piezoelectric transducers under various conditions.

## 1. Introduction

Piezoelectric transducers convert electrical energy into mechanical energy by using the reverse piezoelectric effect. They are widely used in ultrasonic processing, medical engineering, micro-robotics, aerospace, and nanometer-scale positioning systems due to their simplicity of fabrication, high energy output, lightweight, and small size [1,2,3,4,5].

It is well known that the piezoelectric transducer resonates when the driving frequency applied to the driver is the same as the intrinsic frequency of the transducer [6,7]. At the moment of resonance, it has the highest energy conversion efficiency [8]. The common inverter topologies for ultrasonic drivers include full-bridge inverters, half-bridge inverters, and push–pull inverters [9]. Full-bridge inverters are the most commonly used high-power inverters with a high output voltage and high output efficiency [10]. However, full-bridge inverters have four switching devices with high switching losses for large sizes, which are more suitable for high voltage supply. The half-bridge inverter uses two capacitors instead of one bridge arm of the full bridge, reducing the size of the driver [11]. However, the output voltage is only half of the input voltage, and the power utilization is low. Push–pull inverters have the simplest circuit structure. When the input voltage is the same, the output voltage of the push–pull inverter is the same as that of the full-bridge inverter, and only half of the switching devices are used, which reduces the corresponding switching loss and downsizes the driver [12]. Miniaturized and precision drivers have a promising future in applications that require precision operations in confined spaces, such as minimally invasive surgery and micro-robotics. Therefore, the push–pull inverter is particularly suitable for ultrasonic applications for the reason that it reduces energy loss, saves circuit costs, and facilitates integration.

For a high-efficiency system, the piezoelectric transducer must be driven at the resonant point to achieve maximum power transfer [13]. There are many ways to realize frequency tracking based on a push–pull inverter. Martin et al. proposed a directional coupling technique for adjusting signals based on the increase or decrease in reflected power from transducers [14]. However, this method has limitations when the driving frequency is higher than the transducer frequency and requires more control circuits for compensation. Ben-Yaakov et al. proposed a self-regulating push–pull inverter that matches the resonant element through a variable inductor [15]. The frequency can be tracked even if the resonant element and load change. However, the variable inductor requires additional circuits to provide the bias current required by the auxiliary winding, which lowers the power density and overall efficiency [16]. Viguier et al. developed a theory for estimating the frequency of a piezoelectric transducer based on the normal force, using a push–pull inverter to measure the supply voltage and the normal force voltage [17]. However, this architecture is open-loop and cannot stably track the frequency. Liu et al. proposed a push–pull inverter using LLCC matching so that the output voltage amplitude and phase do not vary with the load and frequency [18]. However, the matching parameter selection is complex, and the voltage gain of the matching network is slightly larger than 1, which cannot achieve the boost effect of the matching network. Therefore, a simple and fast real-time frequency tracking strategy based on push–pull topology needs to be developed.

**Figure 1:**
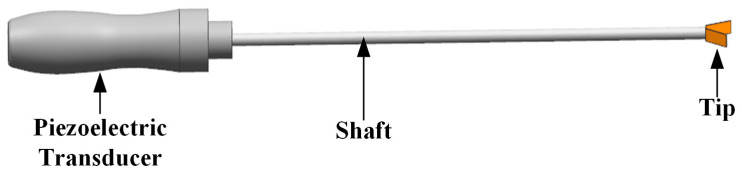
The structure of the ultrasonic scalpel.

Another challenge is to achieve precise displacement amplitude adjustment of the piezoelectric transducer to ensure proper mechanical motion. The ultrasonic scalpel, for example, is shown in Figure 1. The tip frequently contacts different tissues during surgeries. Therefore, precise displacement amplitude control is required over various loads. Fang et al. proposed a parallel resonant frequency tracking and amplitude control scheme based on a transformer ratio-arm bridge [19]. However, this method is only suitable for transformer primary matching and is not applicable for push–pull inverter circuits in the case of transformer secondary matching. Ultrasonic driver circuits should be developed in the direction of miniaturization, low cost, guaranteed accuracy, and versatility, so an improved ultrasonic driver needs to be developed to realize real-time frequency tracking and amplitude control under a wide range of loads while minimizing the driver size and cost as much as possible.

In this paper, a novel driver based on a push–pull inverter is proposed for frequency tracking and amplitude control. First, the driver selects the parallel resonant frequency as the operating frequency of the piezoelectric transducer through theoretical analysis. Second, the driver detects the amplitude and frequency signals of the piezoelectric transducer by a voltage sensing bridge in the case of transformer secondary matching. Then, the overall architecture of the control scheme is introduced, describing the hardware design and control structure of amplitude control and frequency tracking in the case of transformer secondary matching. Finally, the scheme is verified and compared with the conventional scheme regarding amplitude response, load adaptability, and frequency tracking accuracy. In addition, the amplitude control performance is further verified by the USM.

## 2. Theoretical Analysis

### 2.1. Equivalent Circuit Model

**Figure 2:**
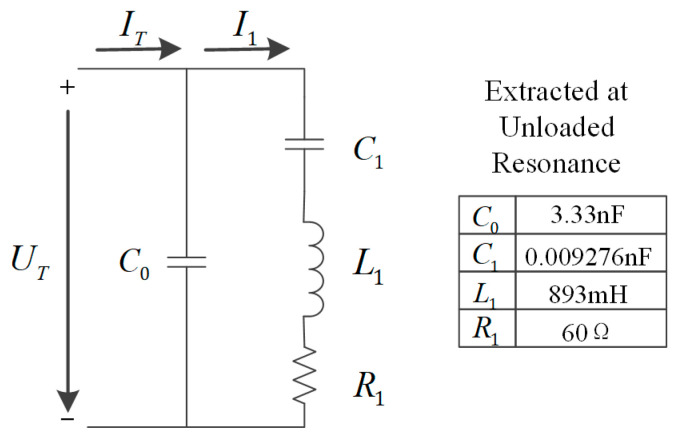
BVD equivalent circuit model of the piezoelectric transducer and the electrical parameters of the piezoelectric transducer (extracted by an impedance analyzer).

The analysis of piezoelectric transducers using equivalent circuit models is a common approach. The Butterworth-van Dyke (BVD) equivalent circuit model [20] of a piezoelectric transducer is shown in Figure 2.

Where $C_{0}$ is the static capacitance of piezoelectric transducers; $C_{1}$, $L_{1}$ and $R_{1}$ correspond to the equivalent capacitance, equivalent inductance, and resistance of the modal stiffness, modal mass, and modal damping, respectively [9]. $I_{T}$ is the excitation current, and $I_{1}$ is the dynamic branch current. The impedance $Z_{S}$ of the equivalent circuit can be expressed by the following formula.

(1) $$Z_{s}=\frac{\frac{1}{j2\pi fC_{0}}\times \left(R_{1}+j2\pi fL_{1}+\frac{1}{j2\pi fC_{1}}\right)}{\frac{1}{j2\pi fC_{0}}+\left(R_{1}+j2\pi fL_{1}+\frac{1}{j2\pi fC_{1}}\right)}$$

The impedance of the dynamic branch $Z_{1}$ can be derived as the following equation.

(2) $${Z_{1}=R}_{1}+j2\pi fL_{1}+\frac{1}{j2\pi fC_{1}\;}\;$$

The piezoelectric transducer has two natural frequencies: the series resonant frequency ($f_{s}$) and the parallel resonant frequency ($f_{p}$), which can be expressed as the following equation.

(3) $$f_{s}=\frac{1}{2\pi \sqrt{L_{1}C_{1}}}\;;\;\;\;f_{p}=\frac{1}{2\pi \sqrt{\frac{L_{1}C_{1}C_{0}}{C_{0}+C_{1}}}}$$

At the series resonant frequency, the dynamic branch impedance $Z_{1}$ is minimized, so the dynamic branch current $I_{1}$ is at its maximum. Physically, $I_{1}$ is proportional to the velocity of the piezoelectric transducer [21]. Therefore, the maximum vibration speed can be obtained. However, when we choose the driving frequency, not only the amplitude should be considered, but also the loss of the piezoelectric transducer and the driving efficiency of the entire system.

Yang et al. pointed out that the loss of piezoelectric transducer depends mainly on the $I_{T}$ and found a significant thermal improvement near the parallel resonant frequency [22]. When the piezoelectric transducer operates at the $f_{p}$, it generates less heat, which can improve its overall performance and reduce the impact of temperature on stability. 

**Figure 3:**
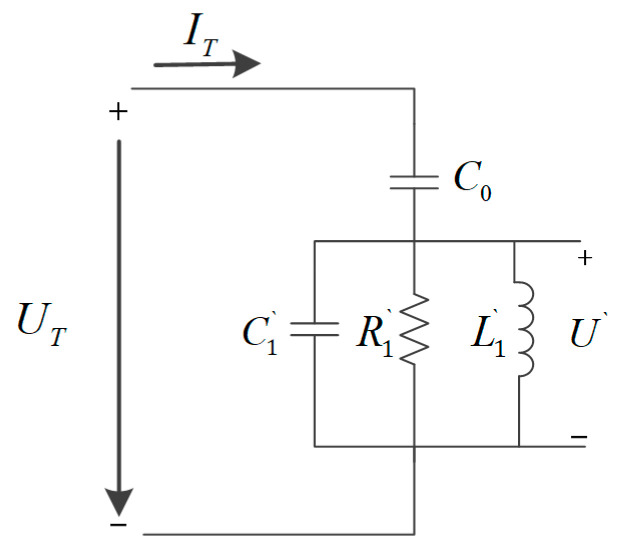
BVD parallel equivalent circuit model.

When the piezoelectric transducer operates at the $f_{p}$, the conventional BVD equivalent circuit model can be converted to a parallel BVD equivalent circuit model [23], as shown in Figure 3.

According to the parallel BVD equivalent circuit model, the impedance $Z_{p}$ of a parallel BVD equivalent can be deduced as the following equation.

(4) $$Z_{p}=\frac{1}{j2\pi fC_{0}}+\frac{1}{\frac{1}{R_{1}^{\prime }}+\frac{1}{j2\pi fL_{1}^{\prime }}+j2\pi fC_{1}^{\prime }}$$

The impedance of the circuit is equal before and after conversion. Therefore, the parallel equivalent circuit model parameters $R_{1}^{\prime }$, $L_{1}^{\prime }$, and $C_{1}^{\prime }$ can be deduced as the following formula.

(5) $$\;\;\left\{\begin{array}{c} R_{1}^{\prime }=\frac{1}{{\left(2\pi f\right)}^{2}C_{0}^{2}R_{1}^{2}}\\ L_{1}^{\prime }=\frac{C_{0}C_{1}/\left(C_{0}+C_{1}\right)}{{\left(2\pi f\right)}^{2}C_{0}^{2}}\\ C_{1}^{\prime }={\left(2\pi f\right)}^{2}C_{0}^{2}L_{1} \end{array}\right.$$

According to Equations (3) and (5), the parallel resonant frequency can be derived as the following equation.

(6) $$f_{p}=\frac{1}{2\pi \sqrt{L_{1}C_{1}C_{0}/(C_{0}+C_{1})}}=\frac{1}{2\pi \sqrt{L_{1}^{\prime }C_{1}^{\prime }}}$$

When the piezoelectric transducer operates at the $f_{p}$, $L_{1}^{\prime }$ resonates with $C_{1}^{\prime }$. According to Equation (4), it can be concluded that the excitation current $I_{T}$ is minimized at the parallel resonant frequency. This also proves that the piezoelectric transducer at the parallel resonant frequency generates smaller losses. Therefore, the $f_{p}$ is more suitable as the excitation frequency of the piezoelectric transducer.

### 2.2. The Extraction of $f_{p}$

The stability of the piezoelectric transducer operation is strongly influenced by the temperature and load characteristics. The resonant frequency decreases as the temperature rises over time [24]. Moreover, the resonant frequency can also be affected due to changes in the load characteristics [25]. Therefore, it is crucial to ensure that the piezoelectric transducer operates at parallel resonant frequencies under different operating conditions.

From the voltage loop of the parallel part of Figure 3, the relationship between the partial voltage $U^{\prime }$ and the excitation current $I_{T}$ can be deduced as the following equation.

(7) $$I_{T}=U^{\prime }\left(\frac{1}{R_{1}^{\prime }}+\frac{1}{j2\pi fL_{1}^{\prime }}+j2\pi fC_{1}^{\prime }\right)$$

**Figure 4:**
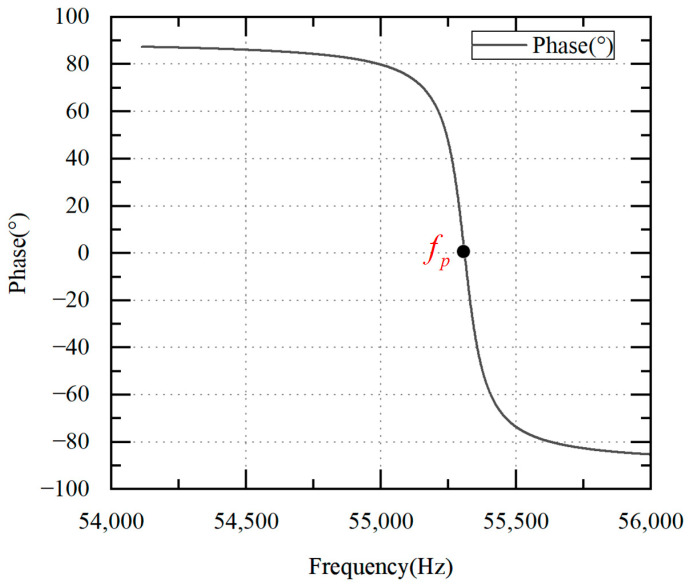
Phase difference–frequency characteristic curve of excitation current and partial voltage.

Taking the ultrasonic scalpel used in this paper as an example, its equivalent circuit model parameters under no load are $C_{0}=3.3\;nF$, $C_{1}=0.009276\;nF$, $L_{1}=893\;mH$ and $R_{1}=60\;\Omega$. The phase–frequency characteristic curves of the partial voltage $U^{\prime }$ and the excitation current $I_{T}$ can be obtained according to Equation (7), as shown in Figure 4.

Figure 4 shows that the phase difference is positive when the $U^{\prime }$ is ahead of the $I_{T}$ and negative when the $U^{\prime }$ is lagging behind the $I_{T}$. The $f_{p}$ can be extracted when the phase difference θ between the $U^{\prime }$ and the $I_{T}$ reaches 0°. Therefore, the piezoelectric transducer can operate at the $f_{p}$ as long as the phase difference between the $U^{\prime }$ and the $I_{T}$ is zero.

### 2.3. Detection of Amplitude

In the BVD equivalent model, the tip velocity v of piezoelectric transducers is linear to the dynamic branch current $I_{1}$ [21].

(8) $$v\propto I_{1}$$

According to Figure 2 and Figure 3, the voltage loop equations of the conventional BVD equivalent circuit model and the parallel BVD equivalent circuit model can be derived, the relationship between the dynamic branch current $I_{1}$ and the partial voltage $U^{\prime }$ is derived as the following equation.

(9) $$\left\{\begin{array}{c} U_{T}\times j2\pi fC_{0}+I_{1}=I_{T}\\ {(U}_{T}{-U}^{\prime })\times j2\pi fC_{0}=I_{T} \end{array}\right.$$

Therefore, the relationship between $I_{1}$ and $U^{\prime }$ is derived as the following equation.

(10) $$I_{1}=-j2\pi fC_{0}U^{\prime }$$

The relationship between the vibration velocity and amplitude of the piezoelectric transducer is shown in the following equation [19].

(11) v=j2πfA

From Equations (8), (10) and (11), the relationship between $U^{\prime }$ and A can be deduced as the following formula.

(12) $$A\propto -C_{0}U^{\prime }$$

Therefore, the amplitude of the piezoelectric transducer can be detected by simply extracting the partial voltage $U^{\prime }$. 

## 3. Implementation of the Proposed Scheme

### 3.1. Voltage Sensing Scheme in Push–Pull Topology

**Figure 5:**
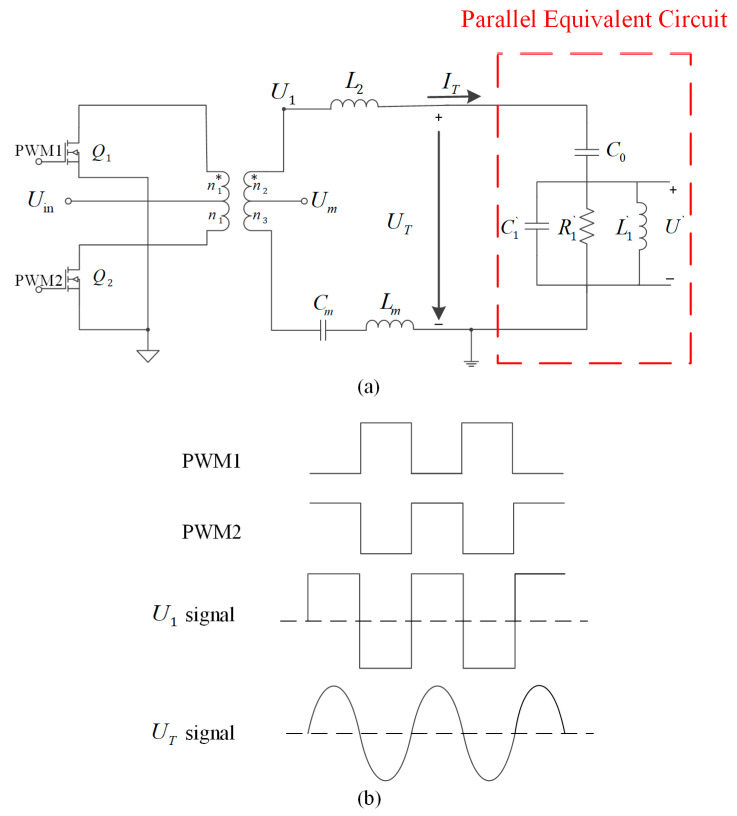
(**a**) Voltage sensing bridge model in push–pull inverter and (**b**) the waveforms of the proposed scheme.

From the previous analysis, it is clear that the partial voltage $U^{\prime }$ is proportional to the amplitude A of the piezoelectric transducer, but the partial voltage cannot be directly measured by the sensor because it is a physical quantity inside the piezoelectric transducer. Therefore, a voltage sensing bridge model based on a push–pull inverter is proposed in this paper, as shown in Figure 5a. It consists of MOSFET transistors $Q_{1}$ and $Q_{2}$, a transformer with taps, matching inductors $L_{2}$ and $L_{m}$, a matching capacitor $C_{m}$ and the parallel equivalent circuit model of the piezoelectric transducer. The MOSFET transistor type is IRFP4227.

Figure 5b shows the waveform of the proposed scheme. Under the action of two PWM waves with a 180° phase difference at the gate, the two switching tubes of the push–pull inverter alternate conduction through the center of the transformer primary, which in turn converts the direct current (DC) into an alternating current (AC). Since the output voltage $U_{1}$ of the push–pull inverter is a square wave, it is necessary to use a matching circuit to filter the square wave into a sinusoidal signal. Therefore, an inductor $L_{2}$ is added to the secondary side of the transformer in series with the piezoelectric transducer to form a matching circuit. The matching circuit filters the voltage waveform and provides impedance matching.

Using a transformer ratio-arm bridge to detect the partial voltage $U^{\prime }$ is easy to integrate with little impact on the electrical circuit [26]. However, the push–pull inverter used in this paper requires the installation of matching inductors on the secondary side of the transformer. Therefore, we propose an improvement based on the transformer ratio-arm bridge, adding a matching inductor $L_{m}$ to eliminate the influence of $L_{2}$.

The voltage loop equation is established from the transformer secondary circuit in the voltage sensing bridge, as shown in Equation (13).

(13) $$\left(U_{m}+\frac{I_{T}}{j\omega C_{m}}+I_{T}j\omega L_{m}\right)\frac{n_{2}+n_{3}}{n_{3}}-\frac{I_{T}}{j\omega C_{m}}-I_{T}j\omega L_{m}=U_{T}+I_{T}j\omega L_{2}$$

$U_{m}$ is the voltage from the transformer tap to ground. Due to the influence of the secondary matching inductor $L_{2}$ and the static capacitor $C_{0}$, it is necessary to set the matching capacitor $C_{m}$ and matching inductor $L_{m}$ to eliminate the influence. It can be noted that when $L_{m}$ and $C_{m}$ are set as the following equations, the tap voltage $U_{m}$ is proportional to $U^{\prime }$.

(14) $$L_{m}=\frac{n_{3}}{n_{2}}L_{2}$$

(15) $$C_{m}=\frac{n_{2}}{n_{3}}C_{0}$$

$n_{1}$, $n_{2}$, $n_{3}$ are the number of transformer winding turns, and the $U_{m}$ can be expressed as the following equation.

(16) $$U_{m}=\frac{n_{3}}{n_{2}+n_{3}}U^{\prime }$$

Therefore, the tap voltage $U_{m}$ can characterize the partial voltage $U^{\prime }$, and consequently, it can represent the amplitude A. Meanwhile, the $f_{p}$ can be detected by using a phase discriminator to obtain the phase difference θ between the tap voltage $U_{m}$ and the excitation current $I_{T}$. This approach makes $U_{m}$ more robust to load variations, ensuring stable frequency tracking and amplitude control under various load conditions.

### 3.2. Hardware Structure

**Figure 6:**
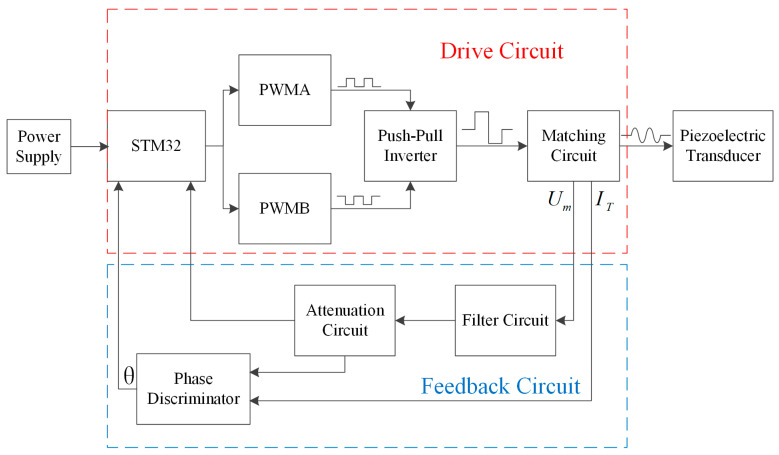
Hardware architecture of the frequency tracking and amplitude control scheme based on the push–pull inverter.

Figure 6 shows the hardware architecture of the frequency tracking and amplitude control scheme based on the push–pull inverter, including the STM32 microcontroller, DC power supply, push–pull inverter circuit, matching circuit, piezoelectric transducer, and feedback circuit.

The stm32 microcontroller sends two-phase pulse width modulated (PWM) signals with a phase difference of 180° to the push–pull inverter. The duty cycle and frequency of the PWM wave are adjusted by the control requirements. The push–pull inverter converts the DC into AC under the control of PWM signals. The output AC square wave is filtered into a sinusoidal wave by a matching circuit, and then the sinusoidal wave can drive the piezoelectric transducer. The amplitude and frequency of drive signals are determined by the frequency and duty cycle of the PWM wave. The feedback signal is the tap voltage $U_{m}$ and the excitation current $I_{T}$. The tap voltage $U_{m}$ is extracted by the voltage sensing bridge. Due to the effect of noise and high harmonics and the fact that the input voltage to the microcontroller is within 3.3 V, it is necessary to preprocess the $U_{m}$. $U_{m}$ first passes through a second-order RLC series bandpass filter circuit, which serves to suppress high-frequency noise and high harmonics. Then, $U_{m}$ is attenuated to within 3.3 V using an attenuation circuit and measured by the microcontroller’s analog-to-digital converter (ADC). The phase difference θ between $U_{m}$ and $I_{T}$ is obtained by a digital phase discriminator. The STM32 microcontroller can detect the amplitude by the processed $U_{m}$ and obtains the $f_{p}$ by the phase difference θ between $U_{m}$ and $I_{T}$ through the phase discriminator. Due to the symmetry of the two-phase signals, only a single-phase signal is demonstrated [27].

### 3.3. Control Structure

**Figure 7:**
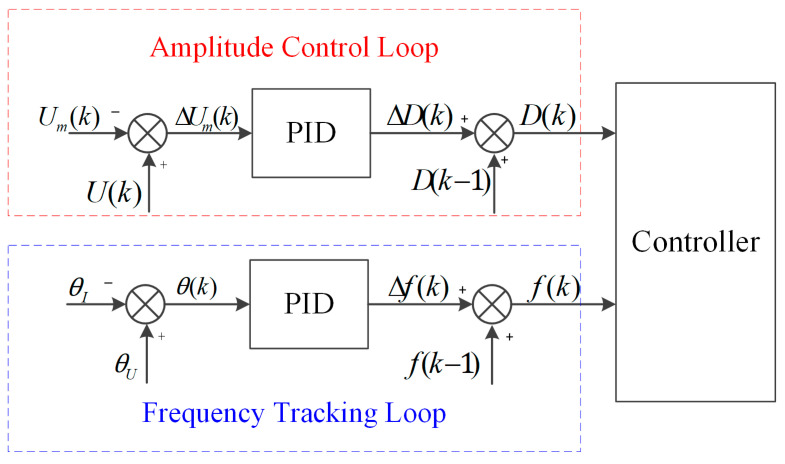
The control structure of proposed scheme.

The control block diagram architecture is shown in Figure 7, including the frequency tracking loop, amplitude control loop, and two PID controllers. The $f_{p}$ and amplitude are detected by processing the feedback signals $U_{m}$ and $I_{T}$. The frequency tracking control loop detects the $f_{p}$ based on the θ between the tap voltage $U_{m}$ and the excitation current $I_{T}$, and adjusts it in real-time to make the phase difference converge to 0°. The purpose of frequency tracking is to make the piezoelectric transducer operate at the $f_{p}$ to ensure maximum output power and energy efficiency.

In the frequency tracking loop, the PID controller can be expressed as

(17) $$\begin{array}{c} ∆f\left(k\right)=K_{p,f}\left[\theta \left(k\right)-\theta \left(k-1\right)\right]+K_{i,f}\theta \left(k\right)\\ \;\;\;\;\;\;\;\;\;\;\;\;\;\;\;\;\;\;\;\;\;\;+K_{d,f}\left[\theta \left(k\right)-2\theta \left(k-1\right)+\theta \left(k-2\right)\right] \end{array}$$

(18) $$\theta \left(k\right)=\theta_{U}-\theta_{I}$$

where $∆f\left(k\right)$ is the increment of the driving frequency, $\theta_{U}$ and $\theta_{I}$ is the phase of feedback signals $U_{m}$ and $I_{T}$, $\theta \left(k\right)$ is the phase difference, and $K_{p,f}$, $K_{i,f}$, and $K_{d,f}$ are the proportional, integral, and differential coefficients, respectively. In this experiment, the values of $K_{p,f}$, $K_{i,f}$, and $K_{d,f}$ are set to 0.3, 0.2, and 0.05 by the trial-and-error method. In a closed control loop for frequency tracking, the frequency increment $∆f\left(k\right)$ is calculated by detecting the phase difference $\theta \left(k\right)$ between $U_{m}$ and $I_{T}$ according to Equation (17), and then by adjusting the driving frequency $f\left(k\right)$ accordingly.

The amplitude control loop is used to keep the amplitude at the target amplitude by changing the duty cycle $D\left(k\right)$ according to the deviation of the target amplitude from the actual amplitude. The PID controller of the amplitude control loop can be expressed as

(19) $$\begin{array}{c} ∆D\left(k\right)=K_{p,D}\left[∆U_{m}\left(k\right)-∆U_{m}\left(k-1\right)\right]+K_{i,D}∆U_{m}\left(k\right)\\ \;\;\;\;\;\;\;\;\;\;\;\;\;\;\;\;\;\;\;\;\;+K_{d,D}\left[∆U_{m}\left(k\right)-2∆U_{m}\left(k-1\right)+∆U_{m}\left(k-2\right)\right] \end{array}$$

(20) $$∆U_{m}\left(k\right)=U\left(k\right)-U_{m}\left(k\right)$$

where $∆D\left(k\right)$ is the increment in the duty cycle, $∆U_{m}\left(k\right)$ is the voltage difference between the target amplitude voltage $U\left(k\right)$ and the measured amplitude voltage $U_{m}\left(k\right)$. $K_{p,D}$, $K_{i,D}$, and $K_{d,D}$ are the proportionality, integration, and differentiation coefficients, respectively. In this experiment, the values of $K_{p,D}$, $K_{i,D}$, and $K_{d,D}$ are set to 0.001, 0.000076 and 0.000001 by the trial-and-error method. In a closed loop for amplitude control, the duty cycle increment $D\left(k\right)$ is calculated by detecting voltage difference $∆U_{m}\left(k\right)$ between the target amplitude voltage $U\left(k\right)$ according to Equation (19) and the measured amplitude voltage $U_{m}\left(k\right)$, and the duty cycle is then updated accordingly. Due to the applicability, simplicity, and robustness of the PID controllers, the piezoelectric transducer can work at $f_{p}$ and provide regulated output levels for different applications.

## 4. Experiment Results

### 4.1. Experiment Setup

**Figure 8:**
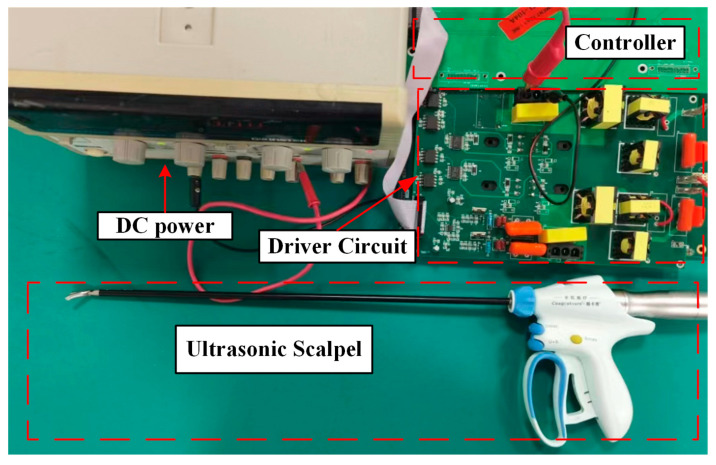
Experimental setup for the ultrasonic scalpel.

**Figure 9:**
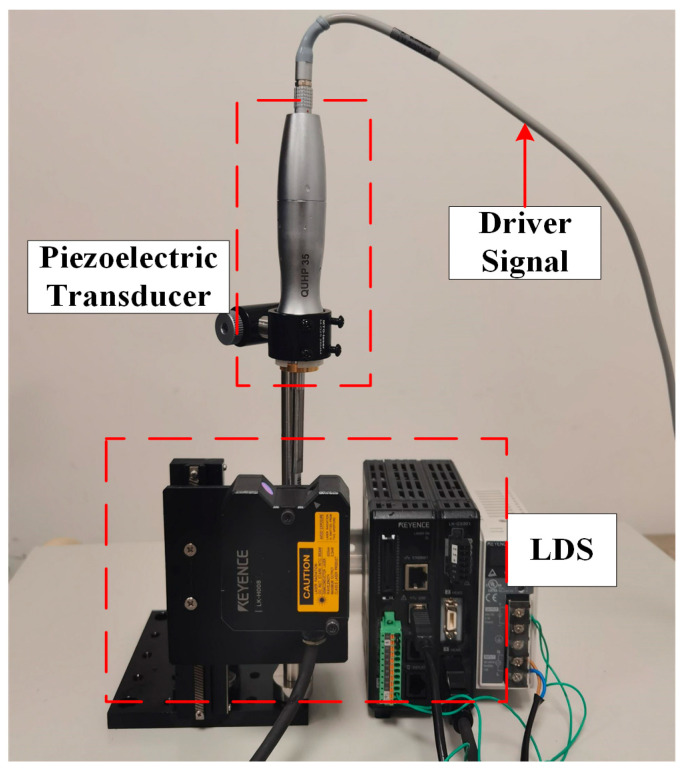
Measurement setup for the piezoelectric transducer.

In this paper, the proposed scheme is verified by the ultrasonic scalpel (BT-US-C55; Bantuo Medical Technology Ltd., Xiangtan City, China). The excitation frequency of the ultrasonic scalpel is 55.5 kHz ± 5.5 kHz. The input voltage was selected as 36 V from a DC power supply. The driver circuit can output two-phase driving voltage, which can be used to provide driving signals for a variety of ultrasonic devices. The experimental setup is shown in Figure 8. The amplitude of the piezoelectric transducer is measured by a laser displacement sensor (LK-H008; KEYENCE, Osaka, Japan). The measurement setup is shown in Figure 9.

The laser displacement sensor (LDS) was set with a sampling frequency of 392 kHz and a measurement range of ±0.5 mm. The data were transmitted to the laptop by the RS-232 interface.

### 4.2. Verification of Amplitude and Feedback Voltage

First, the unloaded amplitude of the piezoelectric transducer and the tap voltage $U_{m}$ is measured to verify the correctness of the scheme. In this experiment, the tap voltage $U_{m}$ is controlled from 0.3 V to 1.0 V by the proposed scheme, and the actual vibration amplitude is measured by the LDS. The LDS has been calibrated by the manufacturer. Each measurement was repeated 5 times.

**Figure 10:**
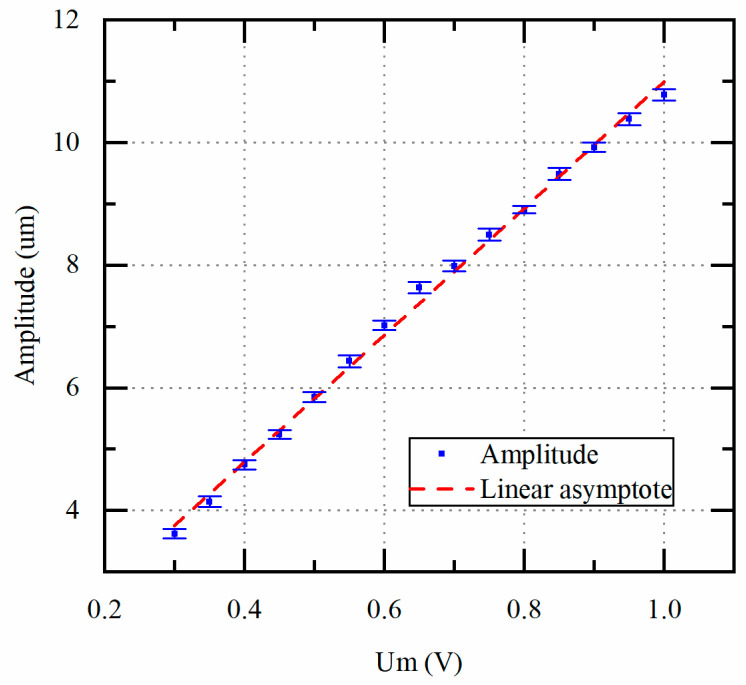
Unloaded amplitude of the piezoelectric transducer vs. tap voltage $U_{m}$.

Figure 10 shows the $U_{m}$ from 0.3 V to 1.0 V with the amplitude from 3.6 μm to 11 μm linearly, which demonstrates that the scheme proposed in Section 2.3 and Section 3.1 can be used to detect the amplitude by the $U_{m}$ in the case of transformer secondary matching. The amplitude here refers only to the amplitude of the piezoelectric transducer. The actual amplitude of the ultrasonic scalpel is further amplified through the shaft.

**Figure 11:**
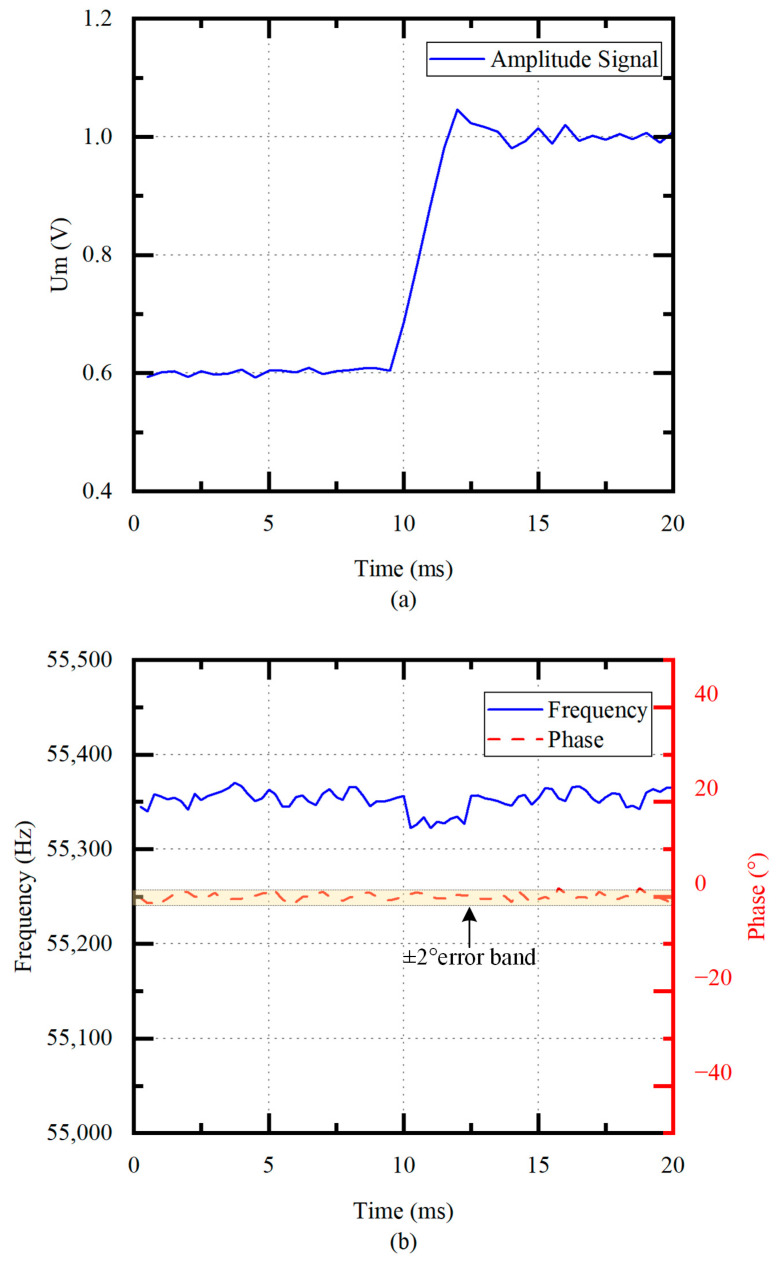
(**a**) Amplitude control process (no load) and (**b**) frequency tracking process (no load).

In order to verify the performance of frequency tracking and amplitude control, the frequency and phase are measured when the tap voltage $U_{m}$ increases from 0.6 V to 1.0 V at unloaded conditions. As shown in Figure 11a, the error of the $U_{m}$ is within ±0.01 V when the system is stable. Figure 11b shows that the drive frequency quickly returns to the $f_{p}$ with the frequency fluctuation of smaller than 50 Hz under frequency tracking. The phase error is within ±2°.

Therefore, this scheme can realize amplitude control and frequency tracking with high efficiency and reliability.

### 4.3. Experimental Results in Glycerin and Chicken Tissue

**Figure 12:**
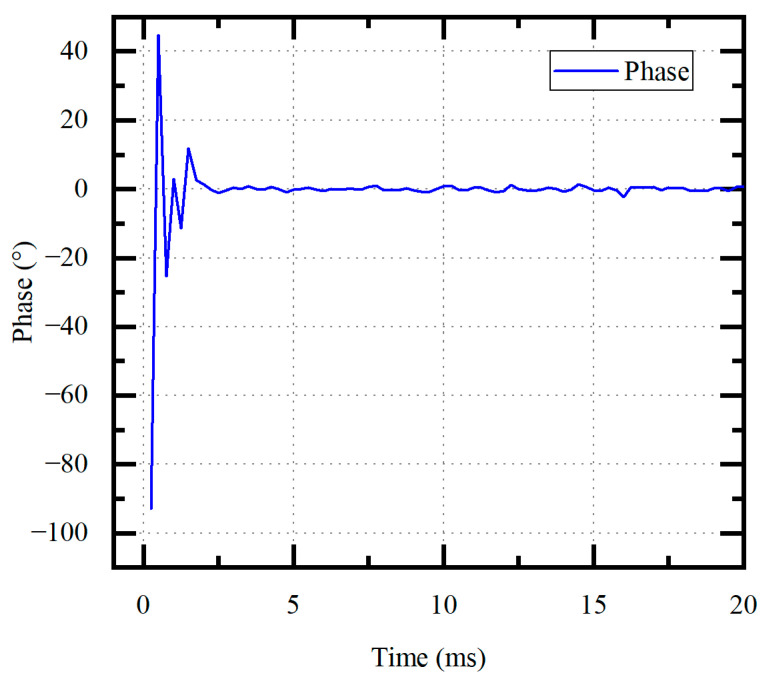
Build-up time of the phase signal.

Transient behavior is also critical to the performance of ultrasonic scalpels. The tip will contact different tissues during surgery, and a fast build-up time is needed to avoid thermal spread. The build-up time is measured by changing the load. Pure glycerol is commonly used to mimic blood and tissues. The ultrasonic scalpel is rapidly inserted into the glycerol to measure the phase signal, as shown in Figure 12. It takes only 3 ms to converge to a stable value, which verifies the fast stability and good transient characteristics of the system under variable load conditions. 

**Figure 13:**
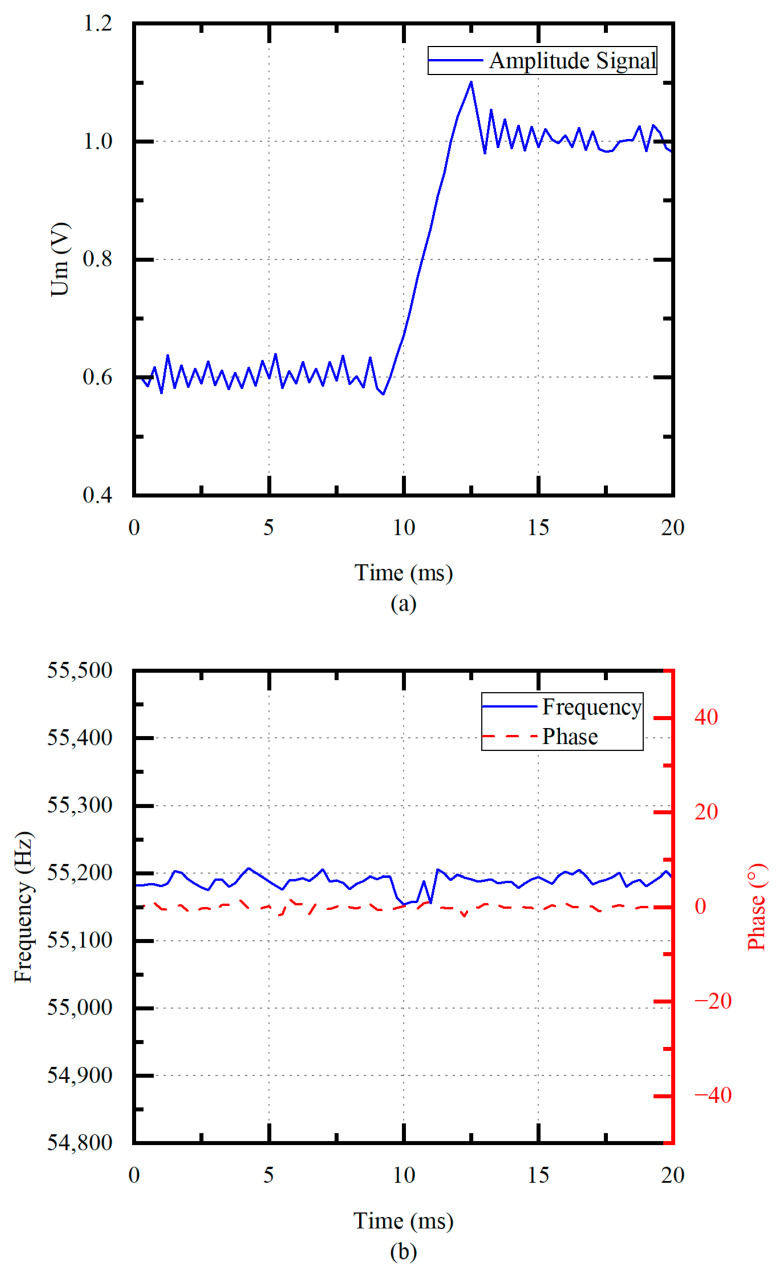
(**a**) Amplitude control process (in glycerol) and (**b**) frequency tracking process (in glycerol).

The system is also tested in load conditions at different amplitudes. The measured tap voltage $U_{m}$, frequency, and phase signals are shown in Figure 13. To simulate the output of different amplitudes in viscous blood, the tip is merged into pure glycerin, and the target amplitude signal $U_{m}$ is set to vary from 0.6 V to 1.0 V. As shown in Figure 13a, the fluctuations of the tap voltage $U_{m}$ are smaller than 0.03 V, which demonstrates reasonable amplitude control under load conditions. Figure 13b shows that the system is quickly adjusted to track the $f_{p}$ by frequency tracking loop under different amplitude, and the $f_{p}$ decreases by 200 Hz compared to the unloaded condition since prolonged operation under load leads to a decrease in the $f_{p}$. In Table 1, the proposed scheme is compared with the conventional ultrasonic scalpel controller [21]. Tracking the $f_{p}$ reduces piezoelectric transducer losses compared to traditional schemes. The large build-up time effectively improves to 3 ms, three times shorter than the conventional scheme. For an unloaded condition, the tracked frequency variation is smaller than 50 Hz. For a glycerin loading condition, the variation range is smaller than 200 Hz. This is because the proposed scheme has better robustness to loads. The inverter topology used in this study is a push–pull inverter, which has the simplest structure compared to a full-bridge inverter. With the same input voltage, a push–pull inverter requires only half the number of MOSFET switching tubes compared to a full bridge inverter to achieve the same output voltage. In addition, the reduction in switching tubes reduces the cost of the circuit, so this scheme is more cost-effective than the conventional scheme. The control scheme proposed in this article can be applied to various applications, such as ultrasonic scalpels and ultrasonic motors, so it has better scalability. Therefore, the proposed scheme has higher tracking accuracy, better driving performance, good feasibility of development and scalability, and a more easily integrated structure.

**Figure 14:**
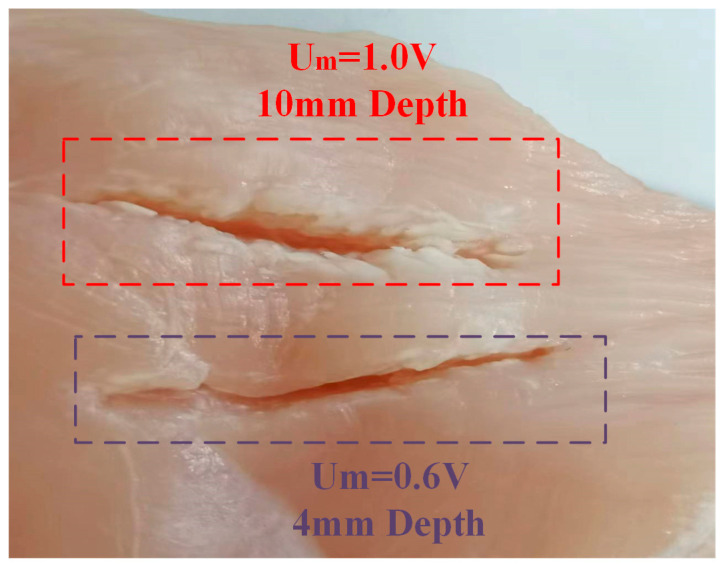
The dissecting function and resulting cutting depths with $U_{m}$ = 1.0 V and 0.6 V.

To test the ultrasonic scalpel capabilities of the amplitude control performance, a piece of chicken breast tissue was cut, as shown in Figure 14. High amplitude provides high output power for clean dissection. When $U_{m}$ = 0.6 V, the cutting depth is 4 mm. When Um is set to 1.0 V as the higher amplitude, the high power results in a deeper cut at 10 mm. The sealing function was measured with low amplitude. 

**Figure 15:**
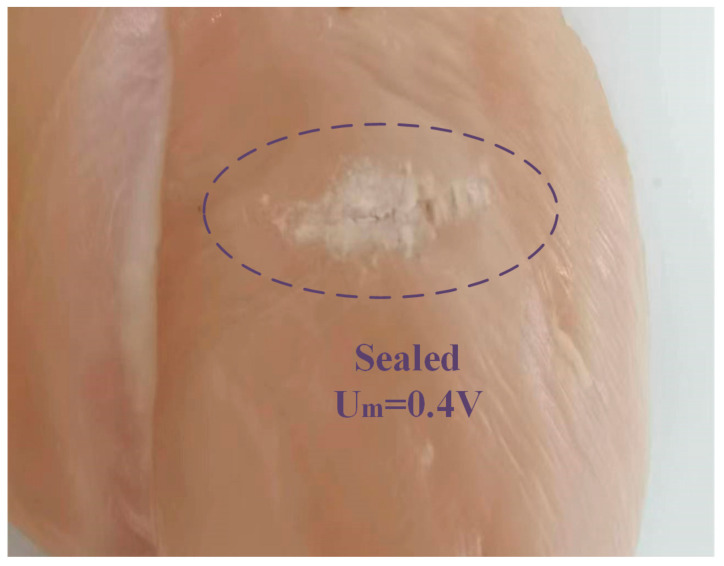
The sealing function setting $U_{m}$ = 0.4 V.

The low mechanical power provided a gentle heat procedure and sealed the tissue. Figure 15 shows a sealing function when $U_{m}$ is set to 0.4 V.

### 4.4. Experimental Results in Ultrasonic Motors

**Figure 16:**
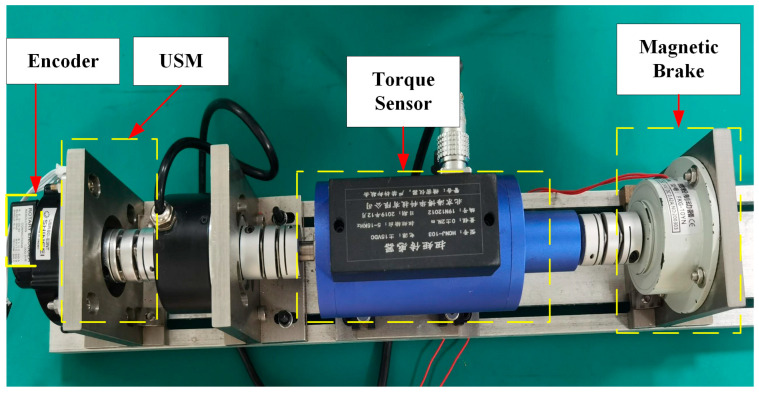
Experimental setup for USM.

The proposed scheme is further validated in USM to verify the amplitude control performance in a wide load range. The designed driver operates on a USM Shinsei-USR60 with a load torque ranging from 0.1 Nm to 0.3 Nm, which quantitatively verifies that the proposed scheme can achieve amplitude control under variable loads. Table 2 lists the characteristics of the USM. The experimental setup for USM is shown in Figure 16. The load torque is provided by a magnetic brake (FKG-10YN; Lanmec electromechanical technology Co., Ltd., Hai’an City, China). An incremental encoder (HEDM-5540-B14; Broadcom Inc., San Jose, CA, USA) detects the velocity. A torque sensor (HCNJ-103; Haibohua Technology Co., Ltd., Beijing, China) with a range of 0 ± 2 Nm is used to measure the output torque and displays the value via attached instrument.

The velocity is linear to the amplitude of the USM due to the slight variation in the working frequency [19]. Therefore, the velocity can represent the amplitude of the piezoelectric transducer in this experiment. The velocity responses of the USM with the amplitude control are measured when the target voltage $U_{m}$ increases from 0.6 V to 1.0 V. The loads are set at 0 Nm, 0.1 Nm, 0.2 Nm, and 0.3 Nm, respectively.

**Figure 17:**
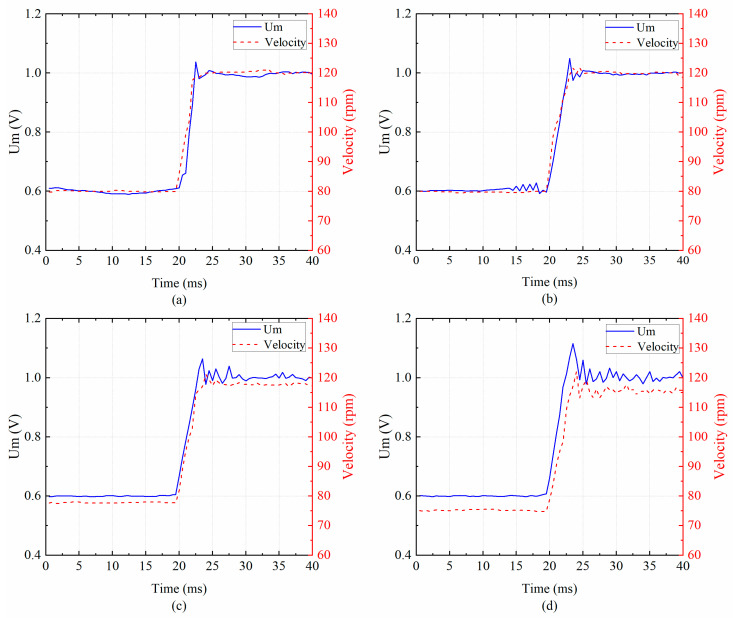
Amplitude control under different loads: (**a**) 0 Nm; (**b**) 0.1 Nm; (**c**) 0.2 Nm; and (**d**) 0.3 Nm.

The experimental results are shown in Figure 17. The amplitude control performance comparison of the proposed scheme and the conventional USM controller is shown in Table 3 [27]. Figure 17 shows the amplitude control scheme can track the target voltage under variable load conditions. Controlling the $U_{m}$ from 0.6 V to 1.0 V under different loads, the velocity response correspondingly ranges from 80 rpm to 120 rpm. Figure 17 shows that when the load torque for the USM changes from 0.1 Nm to 0.3 Nm, the velocity responses decrease from 120 rpm to 114.4 rpm, and the velocity variation is smaller than 5.6 rpm for different load conditions. It can be explained by the contact model of the ultrasonic motor, as an increase in the load torque results in a slight decrease in velocity at the same amplitude [9]. The overshoot of the scheme increases from 1.6% to 5.4%.

Table 3 shows that when the load torque for the TRUSM changes from 0 Nm to 0.3 Nm, the overshoot of the proposed scheme increases from 0.8% to 5.4% and velocity fluctuations increase from 0.96 rpm to 2.26 rpm, while the overshoot of the conventional scheme increases from 0.96% to 6.9% and velocity fluctuations increases from 1.68 rpm to 2.4 rpm. This proves that under variable loads, this scheme based on a push–pull inverter can be used to control the piezoelectric transducer amplitude using the tap voltage and achieve precise mechanical control with good stability and load adaptability while using half the number of switching tubes, reducing driver cost and size.

## 5. Conclusions

In conclusion, this paper proposes a novel driver for the piezoelectric transducer with frequency tracking and amplitude control. Different from conventional methods, this driver is based on a push–pull inverter and extracts amplitude and frequency signals by a voltage sensing bridge in the case of transformer secondary matching. The driver circuit is small and easy to integrate compared to conventional circuits. The designed driver is tested with ultrasonic motors and ultrasonic scalpels to validate the proposed approach. The results show that the proposed scheme can automatically track the frequency and provide proper mechanical functionality regardless of environmental and load variations. The proposed scheme also exhibits better transient performance, achieving fast settling times as low as 3 ms under glycerol loading conditions. The proposed scheme can achieve fast frequency tracking and amplitude control in wide load conditions while using only half the number of switching devices, reducing driver costs and size, which is essential for practical applications. Therefore, the proposed scheme can be applied to ultrasonic applications under variable operating conditions, such as minimally invasive surgery, laser micromachining, and micro-robotic systems.

Due to the complexity of ultrasonic devices, the control strategy is approximately simplified. Adaptive control of the amplitude is required for rapid load changes. In the future, adaptive regulation of output power under rapidly changing loads by frequency tracking and amplitude control will be studied in depth, and future research will integrate circuit components to design monolithic drivers.

## Figures and Tables

**Table 1:** Performance comparison of ultrasonic scalpel.

	This Work	[21]
Resonant Point	$f_{p}$	$f_{s}$
Build-up Time	3 ms	10 ms
Unloaded Frequency Variation	50 Hz	80 Hz
Loaded Frequency Variation	200 Hz	250 Hz
Inverter Topology	Push–Pull Inverter	Full bridge Inverter
The Number of Used MOSFET Switching Tubes in the Inverter	Two	Four
Inverter Complexity	Simple	Complex
Controller Form	Discrete	Discrete

**Table 2:** The characteristics of the USM.

Parameters	Values
Drive Frequency	40–45 kHz
Drive Voltage	130 Vrms
Rated Torque	1.0 Nm
Rated Output	5.0 W
Maximum Velocity	150 rpm

**Table 3:** Performance comparison of USM.

	Proposed Scheme		[27]	
Load (Nm)	Overshoot (%)	Velocity Fluctuation (rpm)	Overshoot (%)	Velocity Fluctuation (rpm)
0	0.8	0.96	0.96	1.68
0.1	1.6	1.44	1.92	1.68
0.2	2.5	1.88	1.94	1.92
0.3	5.4	2.26	6.9	2.4

## Data Availability

Data are contained within the article.
